# CAR-T Cell Therapy

**DOI:** 10.3390/ijms21124303

**Published:** 2020-06-17

**Authors:** Aamir Ahmad

**Affiliations:** Department of Anesthesiology and Perioperative Medicine, University of Alabama at Birmingham, Birmingham, AL 35233, USA; aamirahmad@uabmc.edu; Tel.: +1-205-975-7855

CAR-T therapy has revolutionized the treatment of select hematological malignancies, namely, acute lymphoblastic leukemia and large B-cell lymphomas. Two CAR-T cells-based therapies, Tisagenlecleucel and Axicabtagene ciloleucel, are currently FDA-approved [1]. The therapy involves the harvesting of T cells followed by their genetic modification to express an antigen receptor that is normally not present, thus resulting in the creation of a chimeric molecule—a T cell with the combined specificity of an antibody. This *Special Issue* on “CAR-T Cell Therapy” took a look at the CAR-T cell therapy—its evolution, progress, and promises—as well as its associated challenges and the strategies to overcome these challenges, moving forward. The issue comprised of 4 research articles, 10 review articles and 1 case report.

Currently, approved CAR-T therapies only target hematological malignancies and the next frontier has to be solid tumors. There are several hurdles in the way of adapting this therapy so as to be beneficial against solid tumors. The article by Sacchetti et al. [2] covers this important topic. Further, Th2 immunity mediated by dendritic cells, the antigen-presenting cells, is the subject of discussion in the article by Kumar et al. [3]. This is important given the expression of CD40 ligand, a stimulator of dendritic cells, by CAR-T cells [4]. In addition, claudin 6 expression on dendritic cells and the resulting stimulation of CAR-T cells is evaluated for efficacy of CAR-T therapy against solid tumors [5]. Another target antigen being evaluated is CSPG4, which can potentially bring CAR-T therapy to melanoma, glioblastoma and even breast cancer [6]. It is possible that the key to bringing CAR-T therapy to solid tumors might involve looking at other lymphocytes such as, γδ T, NK, NKT and CIK cells, and not just T-lymphocytes, as discussed by Rotolo et al. [7]. In addition to extending the use of CAR-T therapy against solid tumors, another research area where CAR-T therapy can possibly be effective is against viral infections, such as, HBV infection, as discussed by Boni et al. [8]. 

With the focus on strategies to enhance the efficacy of CAR-T cells, Sitaram et al. [9] describe the utility of evaluating several intracellular proteins which negatively regulate T cell function. Intracellular inhibitory machinery as well as several extracellular receptors are in place to keep immune responses under control and can impact the anti-tumor activity of endogenous T cells as well as the engineered T cells—the CAR-T cells. ROS in the tumor microenvironment is blamed for immunosuppression and the evasion of immune surveillance by cancer cells. Yoo et al. describe a strategy whereby they exploit the high ROS levels to sensitize tumor cells to CAR-T therapy [10]. They achieve this via the use of ROS accelerators that are activated in the presence of high ROS in tumor cells. The combination of CAR-Ts with ROS accelerators seems to work in the in vitro models of leukemia as well as lymphoma and needs to be further tested in clinical settings.

CAR-T therapy in use is CD-19 specific and the relapse is often because of CD-19-negative cells, thus necessitating the evaluation of more antigens to be particularly targeted in relapsed patients. In one of the research articles published in this *Special Issue*, Harrer et al. [11] present proof of concept, using KOPN8 cells, for possible use of CSPG4-specific CAR-Ts against precursor B cell leukemia with MLL translocations. This might be a strategy to not only target the relapse due to CD-19-negative cells, but can be an alternative to CD-19 specific therapy, particularly in tumors expressing CSPG4. As discussed in the article by Abbott et al. [12], identification of alternate and novel tumor target antigens will definitely expand the utility of CAR-T therapy. In a study on these lines, Leong et al. [13] describe results from their study characterizing a novel target to create CARs targeting the ovarian cancer cells in vitro. 

The relapse or the therapeutic success of CAR-T therapy may also depend on the way CAR-T cells are engineered, more specifically on the way they are expanded ex vivo. In the absence of a readily available and optimized CAR-T cells’ production protocol, Stock et al. discuss the various strategies with the aim of improving the overall efficacy of treatment [14]. The engineering of more efficient CARs as well as multi-targeting CARs is discussed in the article by Hughes-Parry et al. [15] which touches upon strategies modifying ecto- as well as endo-domains of CARs. Munter et al. [16] make a case for the use of nanobody technology to efficiently generate CARs constructs to aid clinical testing.

Thus, the advancements in the field of CAR-T therapy have been exciting, to say the least. Clearly, there are challenges ahead that need to be adequately addressed. One challenge, for example, is the better understanding of the host tumor microenvironment, particularly the immune responses, as exemplified by a case study by Funk et al. [17]. Other challenges include making the therapy available for the treatment of many different human cancers, which would involve testing of novel antigens and optimization of CARs to target specific cancers. Another improvement is needed in terms of pricing and affordability as CAR-T therapy remains expensive and cost-prohibitive [18]. Judging by the way this therapy has progressed in recent years, we need to be optimistic about its future expansion and utility.

## Abbreviations

CAR-T	Chimeric Antigen Receptor-T
CIK	Cytokine-induced Killer
CSPG4	Chondroitin Sulfate Proteoglycan 4
HBV	Hepatitis B Virus
MLL	Mixed-Lineage Leukemia
NK	Natural Killer
NKT	Natural Killer T
ROS	Reactive Oxygen Species
Th2	T helper type 2
